# Public data homogenization for AI model development in breast cancer

**DOI:** 10.1186/s41747-024-00442-4

**Published:** 2024-04-09

**Authors:** Vassilis Kilintzis, Varvara Kalokyri, Haridimos Kondylakis, Smriti Joshi, Katerina Nikiforaki, Oliver Díaz, Karim Lekadir, Manolis Tsiknakis, Kostas Marias

**Affiliations:** 1 Institute of Computer Science (ICS), Foundation for Research and Technology - Hellas (FORTH), Heraklion, Crete, Greece; 2 Barcelona Artificial Intelligence in Medicine Lab, Facultat de Matemàtiques I Informàtica, Universitat de Barcelona, Barcelona, Spain

**Keywords:** Artificial intelligence, Breast neoplasms, Magnetic resonance imaging, Public datasets, Software

## Abstract

**Background:**

Developing trustworthy artificial intelligence (AI) models for clinical applications requires access to clinical and imaging data cohorts. Reusing of publicly available datasets has the potential to fill this gap. Specifically in the domain of breast cancer, a large archive of publicly accessible medical images along with the corresponding clinical data is available at The Cancer Imaging Archive (TCIA). However, existing datasets cannot be directly used as they are heterogeneous and cannot be effectively filtered for selecting specific image types required to develop AI models. This work focuses on the development of a homogenized dataset in the domain of breast cancer including clinical and imaging data.

**Methods:**

Five datasets were acquired from the TCIA and were harmonized. For the clinical data harmonization, a common data model was developed and a repeatable, documented “extract-transform-load” process was defined and executed for their homogenization. Further, Digital Imaging and COmmunications in Medicine (DICOM) information was extracted from magnetic resonance imaging (MRI) data and made accessible and searchable.

**Results:**

The resulting harmonized dataset includes information about 2,035 subjects with breast cancer. Further, a platform named RV-Cherry-Picker enables search over both the clinical and diagnostic imaging datasets, providing unified access, facilitating the downloading of all study imaging that correspond to specific series’ characteristics (*e.g.*, dynamic contrast-enhanced series), and reducing the burden of acquiring the appropriate set of images for the respective AI model scenario.

**Conclusions:**

RV-Cherry-Picker provides access to the largest, publicly available, homogenized, imaging/clinical dataset for breast cancer to develop AI models on top.

**Relevance statement:**

We present a solution for creating merged public datasets supporting AI model development, using as an example the breast cancer domain and magnetic resonance imaging images.

**Key points:**

• The proposed platform allows unified access to the largest, homogenized public imaging dataset for breast cancer.

• A methodology for the semantically enriched homogenization of public clinical data is presented.

• The platform is able to make a detailed selection of breast MRI data for the development of AI models.

**Graphical Abstract:**

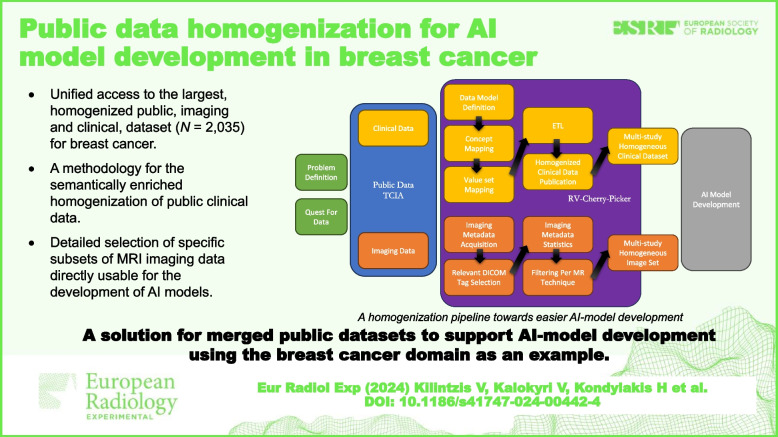

**Supplementary Information:**

The online version contains supplementary material available at 10.1186/s41747-024-00442-4.

## Background

The development of robust clinical artificial intelligence (AI) models heavily depends on access to high-volume and high-quality data. Obtaining an adequate amount of prospective data for AI model development is both costly and time-consuming. A solution to this could be the use of combined retrospective datasets from multiple clinical studies. However, access to homogenous retrospective data is difficult to achieve since each original study had its own specific objectives, and thus, its own data model and acquisition protocol. This makes the acquisition of an integrated homogenous dataset difficult, hampering the secondary usage of the available data. The latter is also an obstacle to using publicly accessible datasets. Publicly accessible datasets are a great solution for fast AI model prototyping, and they also provide the possibility for various AI modelers and researchers to compare the performance of their models without the concern of acquiring shared access to the training data.

In the domain of magnetic resonance imaging (MRI), an additional layer of heterogeneity is introduced among different datasets in terms of the orientation of acquisition (coronal, sagittal, or axial plane), type of MRI sequence used (spin-echo, gradient-echo, inversion-recovery, echo-planar imaging, or any of their variants), fat signal suppression/water excitation options, acquisition conditions (receiver coil selection), etc.

In this direction, basic MRI sequence requirements for optimizing the diagnostic value have been defined by the EUSOBI community [1], constituting an initial image quality indicator. However, several hardware constraints or clinical professionals’ preferences result in a non-negligible degree of heterogeneity among datasets coming from different sites or scanners or even from the same scanner after a sequence of software optimizations. In addition, imaging phenotype is evaluated by radiologists based on the BI-RADS [2] for lesion classification, linking imaging characteristics to tumor grading. Information on the BI-RADS diagnostic category is usually available and compliments the imaging information with a measurable and quasi-objective metric for lesion characterization. Since AI developers heavily rely on high-quality data that ensure high lesion conspicuity, a well-aimed selection of the appropriate cases can be made when a number of sequence-specific metadata are taken into account for building up the specific cohort for a given clinical question.

Apart from the conventional T1-weighted or T2-weighted contrast Digital Imaging and Communications in Medicine (DICOM) series, a breast MRI protocol comprises different techniques, including diffusion-weighted imaging (DWI) and dynamic contrast-enhanced (DCE), which differ from conventional anatomical sequences in the sense that they provide functional information about cellularity and vascularization pattern respectively. Access and selection of the study cases that include images of a specific MRI technique is a preliminary step before developing a certain AI model and that step requires a significant amount of time. Moreover, identifying the number of MRI series of a certain imaging technique in several different datasets might easily highlight whether a public dataset has the required amount of imaging data that are needed to train an AI model.

The aim of the EU-funded RadioVal project [3] is to evaluate the potential of radiomics to predict response to neoadjuvant chemotherapy in breast cancer patients, using AI. The project leverages large imaging repositories and implements a multifaceted evaluation of radiomics AI tools in eight hospitals across Europe and beyond, to test their technical robustness, algorithmic fairness, as well as usability and acceptance in clinical settings.

For developing preliminary AI models, publicly available datasets were primarily explored, identifying five promising datasets available by The Cancer Imaging Archive (TCIA). However, these datasets were heterogeneous requiring a significant effort for their harmonization. The resulting dataset comprises five individual datasets, with information about 2,035 subjects with breast cancer. We present the process followed to (a) harmonize clinical data by developing a common data model and a repeatable, documented extract-transform-load (ETL) process [4]; (b) uniformly expose them, following the Findability, Accessibility, Interoperability, and Reuse (FAIR) principles [5], enabling data selection and filtering; and (c) extract relevant information from the DICOM headers of the corresponding imaging series, enabling selection and downloading of imaging data subsets with specific characteristics. Subsequently, we present “RV-Cherry-Picker,” a free-to-access public tool that provides access to the largest homogenized and semantically enriched dataset with imaging and clinical data publicly available for breast cancer.

## Methods

For developing preliminary AI models for the prediction of treatment response in breast cancer, the RadioVal consortium first identified the public datasets available online. However, to be used for modeling tasks, the datasets needed to be harmonized and integrated.

**Fig. 1:**
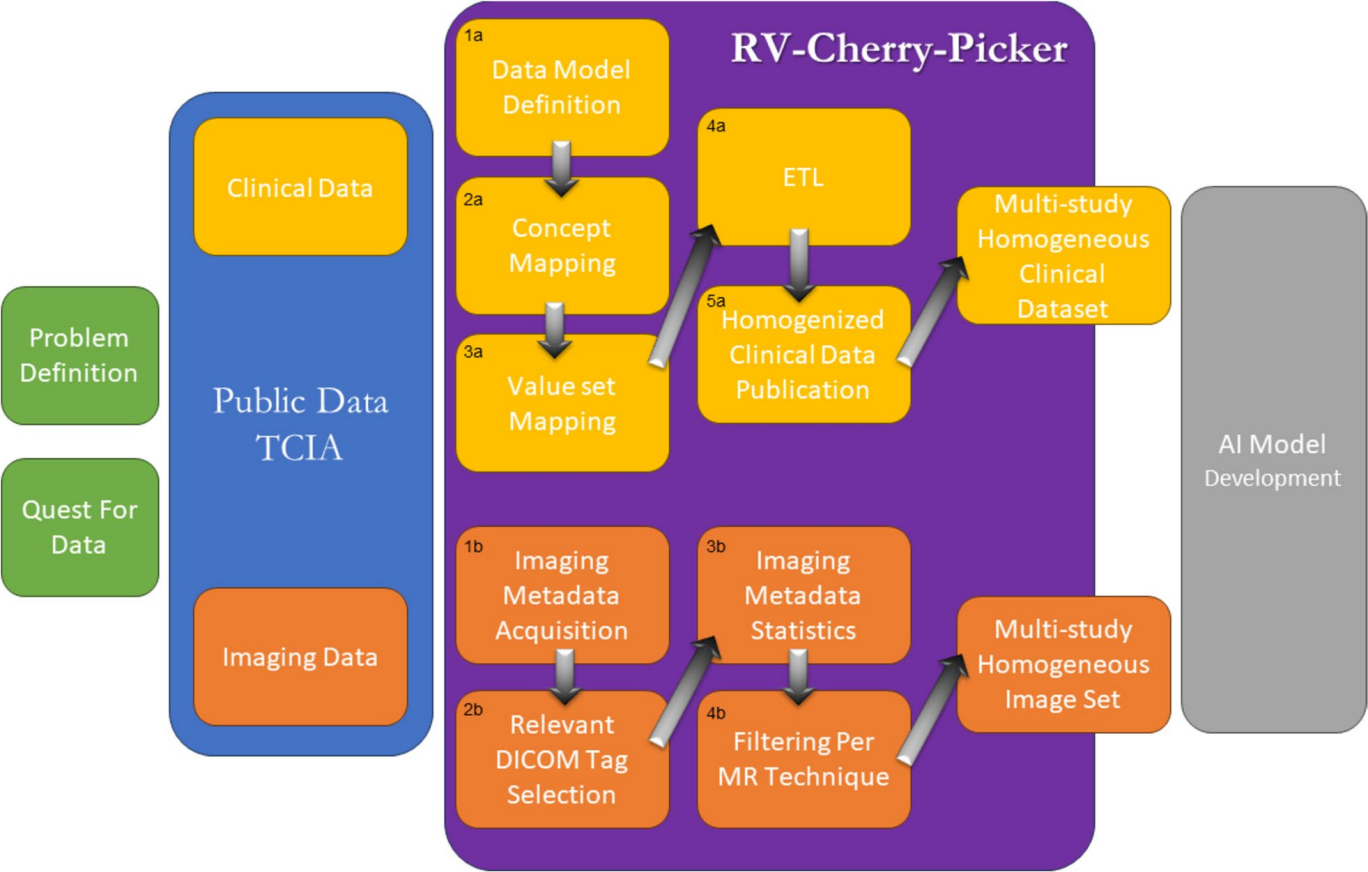
Data homogenization pipeline

The pipeline that was followed for harmonizing and exposing both clinical and imaging data is presented in Fig. 1. Regarding the clinical public data, upon the selection of the publicly available datasets, a data homogenization pipeline was followed that included (a) the definition of a common data model that describes and unifies them (Fig. 1 (1a)); (b) the identification of the corresponding semantic annotations (Fig. 1 (2a–3a)); (c) the ETL process which was applied for each one of the datasets for converting the initial data into the common data model defined (Fig. 1 (4a)); and (d) publication of the FAIRified homogenized dataset available through the RV-Cherry-Picker tool (Fig. 1 (5a)). Regarding imaging data, by leveraging TCIA application programming interface (API), the corresponding imaging metadata was retrieved (Fig. 1 (1b)) and was examined for the presence of the relevant DICOM tags (Fig. 1 (2b)). This process enabled the generation of statistics per public dataset (Fig. 1 (3b)) and the deployment of the graphical tool for the targeted selection of imaging series acquired via a specific MRI technique (Fig. 1 (4b)).

In the following paragraphs, we describe the aforementioned steps in detail for both data selection, homogenization of clinical data, and enhanced selection of imaging data.

### Data selection

The datasets were identified and retrieved through TCIA, a service that deidentifies and hosts a large publicly available archive of medical images of cancer [6]. TCIA provides clinical data in the form of downloadable comma-separated values (CSV) files along with a graphical tool for selecting and downloading imaging studies per subject or study.

Since RadioVal project’s domain of interest is breast cancer, the datasets that we considered in the context were the following:

- I-SPY 2 Breast Dynamic Contrast-Enhanced MRI Trial (ISPY2) [7]
- Dynamic contrast-enhanced magnetic resonance images of breast cancer patients with tumor locations (Duke-Breast-Cancer-MRI / DUKE) [8]
- Multicenter breast DCE-MRI data and segmentations from patients in the I-SPY 1/ACRIN 6657 trials (ISPY1) [9]
- The Cancer Genome Atlas Breast Invasive Carcinoma Collection (TCGA-BRCA) [10]
- Single-site breast DCE-MRI data and segmentations from patients undergoing neoadjuvant chemotherapy (Breast-MRI-NACT-Pilot) [11]

The selected clinical datasets include an arbitrary number of concepts, based on the original study’s requirements and they also have corresponding imaging MRI data. Generally, these clinical concepts correspond to demographic information (*e.g.*, age, ethnicity), clinical and/or tumor pathology and treatment information (*e.g.*, tumor hormone receptor status, TNM stage), and outcome information (*e.g.*, last follow-up date, response to treatment).

For most of these concepts, the corresponding value set is either a number or a value from a predefined list. Quite common is the use of binary (0 or 1) values corresponding to no/yes as a value set while there are also cases where either the field is a concatenation of values or the field contains additional text apart from the expected value (*e.g.*, for tumor position, instead of the expected by the study protocol “L 2,” signifying tumor in left breast at clock hour 2, the field contains “L 2 with calcs”).

Additional information (not explicitly defined in the corresponding CSV file) can be derived from the original study design. For example, in Breast-MRI-NACT-Pilot, all study subjects had undergone adjuvant radiotherapy, or in all ISPY2 study subjects, cyclophosphamide was administered apart from the medication regimen described in the corresponding CSV file.

Lastly, the comment fields in the CSVs were examined to identify possible hidden information, which was considered during the conversion of the dataset. For example, subject 27 in Breast-MRI-NACT-Pilot had the comment “declined standard-of-care post-surgery radiation and hormonal treatments” which resulted in a case-specific definition of the adjuvant therapy fields (*i.e.*, adjuvant.hormone_therapy and adjuvant_radiotherapy were set to “NO”).

In Table 1, the number of distinct subjects in the original CSV files of the public datasets is presented. These files include data corresponding to 2332 study subjects and 251 domain concepts, excluding case identification codes. Note that the number of subjects per dataset varies from around 1,000 in one dataset (*e.g.*, ISPY2) to only 64 subjects in another (*e.g.*, Breast-MRI-NACT-Pilot), and the number of concepts and the granularity of information provided by each dataset varies as well (*e.g.*, ISPY2 has only 9 concepts *versus* 110 concepts in TCGA-BRCA).

**Table 1:** The public datasets that were used along with the number of cases and concepts per case they included in the corresponding clinical data comma-separated values file

Dataset	Number of subjects	Number of concepts	Number of MRI series
ISPY2	985	9	9,391
Duke-Breast-Cancer-MRI	922	74	5,034
ISPY1	221	17	6,464
TCGA-BRCA	140	110	1,858
Breast-MRI-NACT-Pilot	64	41	1,846

*MRI *Magnetic resonance imaging

The selected public datasets also have corresponding MRI DICOM files of different MRI techniques such as DCE or DWI or different orientations (axial, sagittal, or coronal). The number of MR imaging series per dataset is presented also in Table 1.

### Homogenization of clinical data

Definition of the common data model and semantic annotations. The definition of a common data model refers to the selection of concepts to be included, as well as to the selection of possible values for each one of them. The aim of this process, to ensure high reusability through high semantic coherence and completeness, is to produce a single dataset including only scalar (numeric) fields and nominal (categorical) fields that are coded, using codes from standardized vocabularies, *i.e.*, concept identifiers (IDs).

The set of standardized vocabularies that was used in the harmonization process is based on the OMOP [12] standardized vocabularies representing clinical concepts which can be accessed by the ATHENA (Accessible Terminology, Health Information, and Navigation) web-based tool [13] developed by the National Library of Medicine. These vocabularies include standard terminologies such as SNOMED-CT [14], LOINC [15], and UMLS-RxNorm [16] as well as a set of specialized vocabularies, such as the HemOnc Vocabulary [17] and the Cancer Modifiers ontology [18] for representing concepts in the oncology domain. For our purposes, we used ATHENA to browse the various standardized vocabularies while mapping each dataset concept to the corresponding OMOP standardized concept.

For choosing the concepts to include, an analysis was conducted on the four larger datasets (namely, DUKE, ISPY2, ISPY1, and TCGA) collectively, to ascertain the semantics associated with the concepts in each dataset. Although there are many relevant concepts in the breast cancer domain, we examined only concepts originally included in the selected datasets. The main criterion that was used to include a concept in the common data model was to identify the same concept in at least two of the original datasets. The rationale behind this decision was twofold, first, to acquire a dataset with as few as possible missing values and, second, to acquire a dataset that includes more cases having the specific concept defined than any single, original dataset. The latter is a key requirement, set to enhance the added value of the resulting dataset. The process included taking one by one the concepts of each dataset and finding corresponding concepts in the other datasets. The corresponding concepts were either synonymous concepts with the same or similar value sets or a combination of concepts that could be used to evaluate the concept of the common data model. For example, the common data model concept tumor hormone receptor status (OMOP:4,160,341) is part of the ISPY2 dataset as “HR” and as “HR Pos” in ISPY1, although it is not explicitly defined in the DUKE dataset, the fields, “ER,” corresponding to “Status of estrogen receptors of neoplasm” (OMOP:40,481,986), and “PR” mapped to “Status of progesterone receptors of neoplasm” (OMOP:40,481,987) can be used to calculate the appropriate value for all DUKE subjects.

This process was performed also in reverse, *i.e.*, in one dataset a concept was described with semantics that required a generic value-set while in others there was detailed information. In that case, we have decided to calculate the generic values from the detailed information and include both in two distinct concepts in the common data model. For example, in the ISPY2 dataset, neoadjuvant chemotherapy was defined in the field “Arm” using the administered drug name while in DUKE the corresponding “Neoadjuvant Chemotherapy” field was binary. To retain as much information as possible and provide access to as many cases as possible we defined two fields: “neoadjuvant_chemotherapy” and “neoadjuvant_chemo_medication” both mapped to Neoadjuvant chemotherapy (OMOP:44,808,409). For the first, yes (OMOP:4,188,539)/no (OMOP:4,188,540) were defined as value sets, and for the second, single or multiple codes referring to the specific medication were used.

Once the concepts of the common data model and their semantics were clarified, the concepts were mapped manually to OMOP IDs. The selection of the specific concept ID was performed considering the following set of rules:

1. Select the concept that matches the most to the semantics of the concept of the original dataset instead of the specific textual string (“conceptual match VS literal match”). This rule apart from the concept name refers also to the selection of the concept that matches the concept’s class as it is provided by the referenced terminology, *e.g.*, ER was mapped to “Status of estrogen receptors of neoplasm” (OMOP:40,481,986) which is a SNOMED—Observable entity, instead of “Estrogen receptor” (OMOP:4,051,798) which is a SNOMED – Substance.
2. Select concepts of well-known and commonly used terminologies. When possible, we tried to map the concepts to SNOMED, ICD, LOINC, and RxNorm, and if not possible, we then selected from other more disease-specific terminologies such as the Cancer Modifier terminology.
3. Select concepts marked as Valid and Standard over those marked invalid or non-standard assuming the semantics of the valid/standard concepts are adequate;
4. Finally, if more than one concept is still applicable, select the one with the most synonyms to enrich the defined semantics.

Regarding the value sets, the core decision had to do with the use of values with detailed semantics instead of codes of generic values (*i.e.*, yes/no, positive/negative) where this was possible. For example, for the concept “Status of estrogen receptors of neoplasm,” the values were mapped to “Estrogen receptor positive tumor” (OMOP:4,167,696) and “Estrogen receptor negative neoplasm” (OMOP:4,261,933) instead of the “yes/no” used in the original datasets. This decision enhances the semantics of any subset of the homogenized dataset since the value for a specific case encompasses all the meaning without the need to be used in conjunction with the concept. Also, since all possible values were present at the time of conversion, no missing values are present in the final homogenized dataset. In cases where the original dataset had missing or not confirmed values, the concept ID of “Not provided” (OMOP:763,013) was used.

A common challenge was to handle concepts that are equivalent among datasets but whose value sets included terms in different levels of detail and all the terms (*i.e.*, generic and specific) could be mapped to OMOP IDs. An example of such a case is the value sets for the concept “pN category” (OMOP:4,161,174) of the pathological TNM classification in the DUKE and TCGA-BRCA datasets. In the first, the value set is numerical from -1 to 4, with -1 referring to the “NX category,” while in the second it is categorical/text including sub-categories (*e.g.*, N1a, N0 (i-)). In this case, as in other similar cases, we have mapped all possible values of both value sets into distinct codes and thus, the value set of the concept in the homogenized dataset includes both the detailed and the more generic codes. This approach retains all the information that is included in the original dataset and at the same time is easy to recode the field using only the generic value set if this is needed.

For the concepts whose original values were combinations of values (*e.g.*, neoadjuvant chemotherapy medication), each distinct possible value was mapped to an OMOP ID, and the resulting field was defined as a concatenation of the specific concept IDs delimited using the vertical bar. This decision resulted in one field/column per concept in the resulting dataset instead of having multiple columns (*i.e.*, first medication, second medication) and many empty values.

For certain fields, the value set in the original dataset required cleaning prior to any conversion/mapping. This was possible because, in the original data request form, the answer was requested in a free text field and the data provider did not adhere fully to the corresponding instructions. Such a field was the DUKE’s “tumor position”. In the original dataset apart from the expected clock face locations there are also definitions in arbitrary detail, for example, “L 11:30” or “R4-5” and additional text such as “L with calcs” resulting in 259 different field values in the 922 cases included in the dataset. Since information regarding the tumor location was also present in the TCGA-BRCA in the “icd_o_3_site” field, we have selected to map both datasets to the concept “Anatomic location of neoplasm” (OMOP:4,135,405) and use as the value set the OMOP IDs that refer to the relevant concepts from International Classification of Diseases for Oncology, 3rd Edition (ICD-O-3).

### Dataset conversion through an ETL process

The original datasets, as discussed above, were either in Excel (Microsoft Corporation, Redmond, WA, USA) or in CSV format. Instead of performing the conversion of these datasets to the common data model (CDM) manually, we prepared the necessary ETL scripts and documented the procedure so that this operation could be repeated by anyone. The major advantage of this decision against the easier one-off approach is twofold. Firstly, the whole conversion pipeline can be repeated in case of an identified conversion error at any time point without the need to perform several manual steps from scratch. Secondly, this approach provides the possibility to include additional concepts from the existing datasets into the CDM in case of either expansion of the available public datasets that could lead to the inclusion of additional concepts (*i.e.*, concepts that were now omitted from CDM due to the fact that they were present in only one dataset) or use of an in-house dataset which encompasses different concepts from those in our CDM that are included in one of the original datasets.

We have used the Statistical Package for Social Science (SPSS, IBM Corp, Armonk, NY, USA) for the conversion of the datasets. The SPSS scripts are publicly available here [19] for reuse or review. In case additional preparatory steps were required (*e.g.*, removal of specific top rows in Excel to assist auto import from SPSS), those instructions accompany the scripts.

### Enhanced selection of imaging data

In addition to standardizing clinical data, it is essential to have accessible information about imaging techniques and acquisition characteristics identified through specific DICOM tags. The acquisition of the imaging metadata for each image series in a specific public dataset is a two-step procedure. Initially, the NBIA Advanced REST API [20] is queried to retrieve all imaging series that are part of the specific public dataset, and then, one request query per series in the response must be performed to acquire the series’ DICOM tags. This process may require significant time depending on the size of the dataset; thus, the appropriate caching mechanisms were employed to allow faster operation.

Regarding grouping per MRI technique, a set of DICOM tags was selected, and for each dataset, the number of series in various meaningful combinations of these tags was calculated. The set of DICOM tags of interest is presented in Table 2. Moreover, since in public datasets images have commonly undergone anonymization, some DICOM tags that would help identify the imaging technique could be missing, *e.g.*, number of temporal positions. To overcome this, DICOM tags defining technical setup information may be used to safely derive imaging techniques, *e.g.*, the flip angle.^1^ Moreover, DWI, in technical terms, can be described as an echo-planar-based acquisition, acquired in a two-dimensional mode and repeated with different diffusion sensitivity values to acquire the quantitative apparent diffusion coefficient maps. Additionally, for the DWI sequence, the necessary DICOM tags to complement the description were diffusion directionality and diffusion *b*-value. Only one private tag was used to help identify the DWI series (General Electric, 0043,1039). These scanners perform unique ordering of the different *b*-value acquisitions in the series, resulting in apparent diffusion coefficient miscalculation if this is ignored.

**Table 2:** The DICOM tags of interest

DICOM tag	Description
(0018, 0020)	Scanning sequence
(0018, 0023)	MRI acquisition type
(0020, 0037)	Image orientation (patient)
(0018, 0024)	Sequence name
(0008, 103E)	Series description
(0008, 0008)	Image type
(0018, 1314)	Flip angle
(0018, 9075)	Diffusion directionality
(0043, 1039)	Private tag for diffusion *b*-value
(0018, 9087)	Diffusion *b*-value
(0020, 0105)	Number of temporal positions

## Results

In this section, we provide an overview of the results of our activities, which is a homogenized dataset promoting research in the domain and a tool enabling the exploration of this dataset.

### The RV-Cherry-Picker dataset

**Fig. 2:**
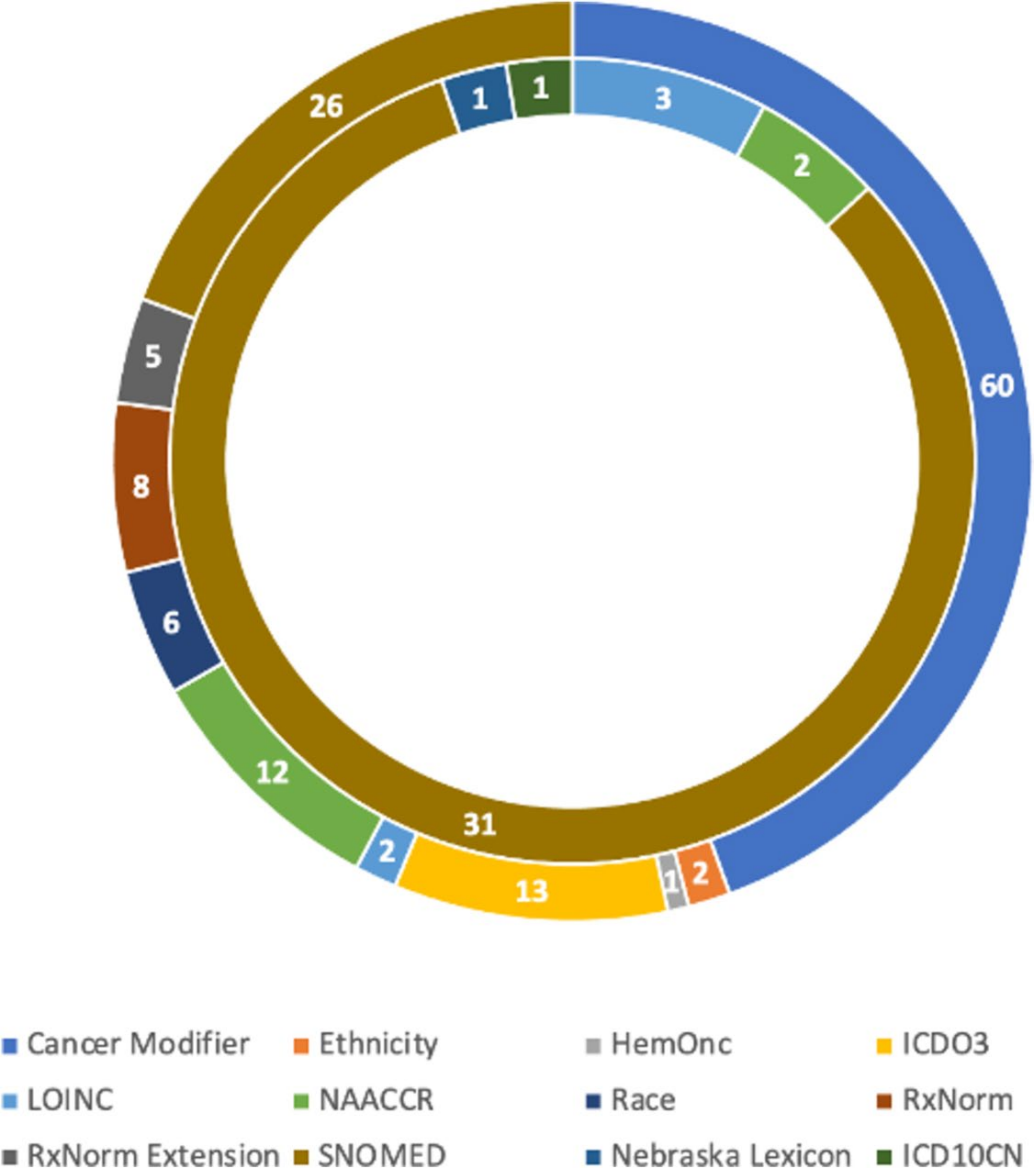
Usage of each vocabulary for concept mapping (inner arc) and value-set mapping (outer arc)

The resulting homogenized dataset includes 2,035 subjects from 5 public datasets. The homogenized clinical dataset describes 38 coded clinical concepts using 135 coded values from 12 standardized vocabularies. In Fig. 2, the vocabularies used both for the coding of concepts (inner arc) and for the coding of the value set (inner arc) are presented. For the concepts, the one mainly used with 31/38 concepts mapped to codes originating from it was SNOMED, while for coding the values, the Cancer Modifier and SNOMED with 60 and 26 values respectively were those used more.

In Table 3, the composition of the homogenized clinical dataset is presented. The first two columns present the final number of subjects that were included in the homogenized dataset along with the corresponding percentage of the original dataset are presented. Then, the number of concepts of the homogenized dataset that were mapped to the study-specific concepts of the original dataset and the corresponding percentage are presented. It must be noted that for the ISPY2 dataset, 12 concepts were coded using the information described in the original 9 concepts (see Table 1) resulting in 133% in the last column. This was a result of the concept selection methodology retaining both detailed and generic concepts.^2^

**Table 3:** Subjects and concepts included in the homogenized dataset

Dataset	Subjects included		Mapped concepts	
	**Number**	**Percentage**	**Number**	**Percentage**
ISPY2	719	73%	12	133%
Duke-Breast-Cancer-MRI	922	100%	36	49%
ISPY1	221	100%	14	82%
TCGA-BRCA	139	99%	27	25%
Breast-MRI-NACT-Pilot	64	100%	23	56%

The 38 selected and coded concepts, their mapping to OMOP concepts, and the presence of each concept in the original dataset are presented in Table 4. The concepts are organized into conceptual groups (*e.g.*, demographic information, pathology report).

**Table 4:**
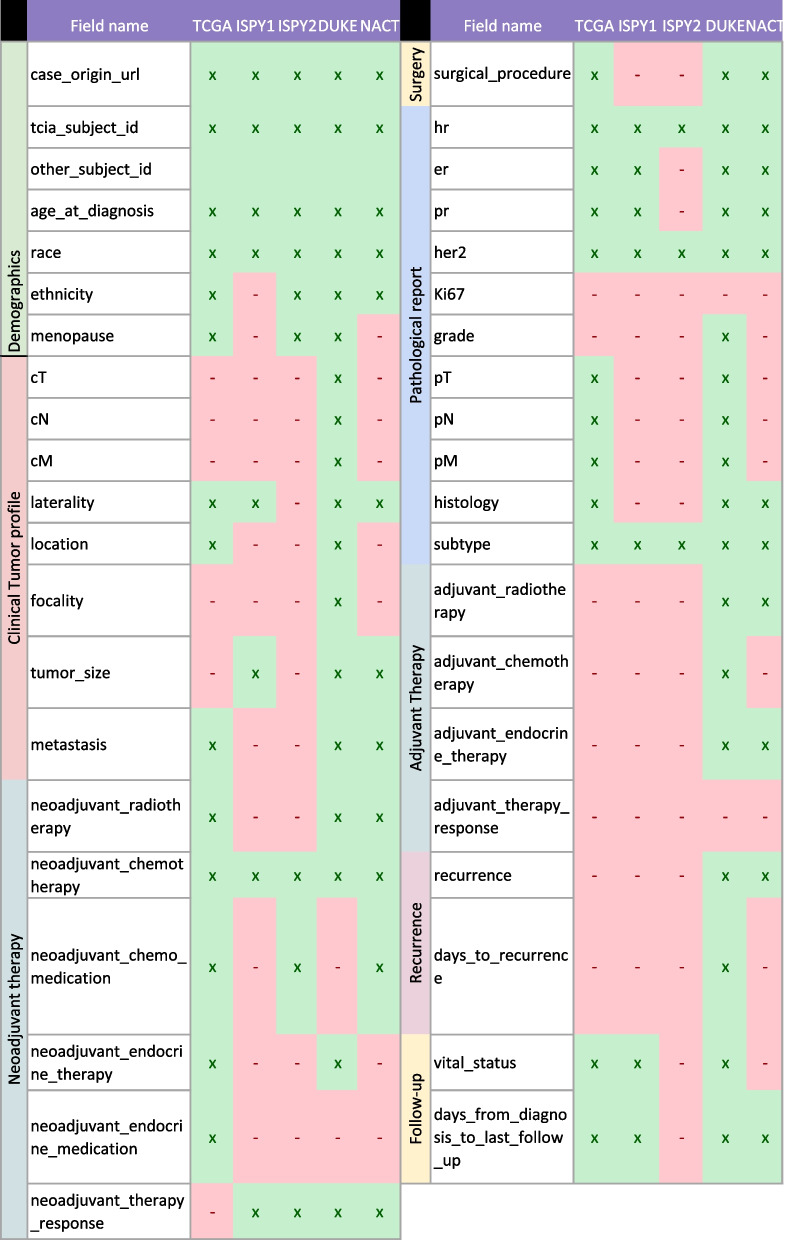
List of included concepts with corresponding OMOP ID and response to the original dataset

### RV-Cherry-Picker tool

**Fig. 3:**
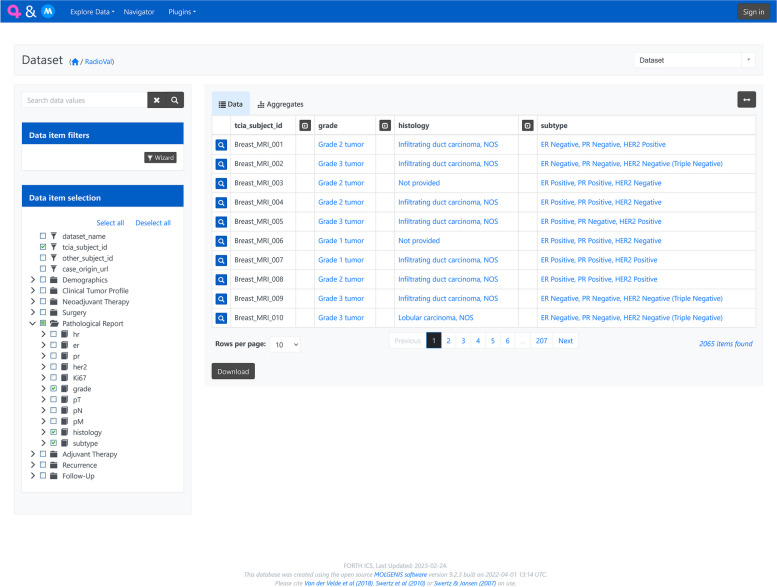
RV-Cherry-Picker clinical

RV-Cherry-Picker is deployed and it is publicly accessible [21] enabling exploration of the homogenized dataset. To provide public FAIR-based access to the homogenized and semantically enriched clinical data set, the Molgenis [22] metadata catalog was used. Molgenis provides two ways of accessing the available information. First, there is a user-friendly interface that allows users to easily filter, search, and sort information according to their needs and specific use cases, allowing download of the result in CSV or Excel format, and second, a set of APIs to retrieve data programmatically based on their own needs. A screenshot of the deployed browser for harmonized open clinical datasets for Breast Cancer (RV-Cherry-Picker clinical) is presented in Fig. 3.

For the second part of RV-Cherry-Picker (Public DICOM cherry picker), a lightweight standalone web application was developed that implements the acquisition, caching, and grouping of the imaging series metadata, and provides the web interface that enables browsing the information per public study and selecting an identified subset of the data. Subsequently, the Public DICOM Cherry Picker generates, for the selected subset of MRI images, the specific manifest file to be used by NBIA Retriever [23] for image downloading.

**Fig. 4:**
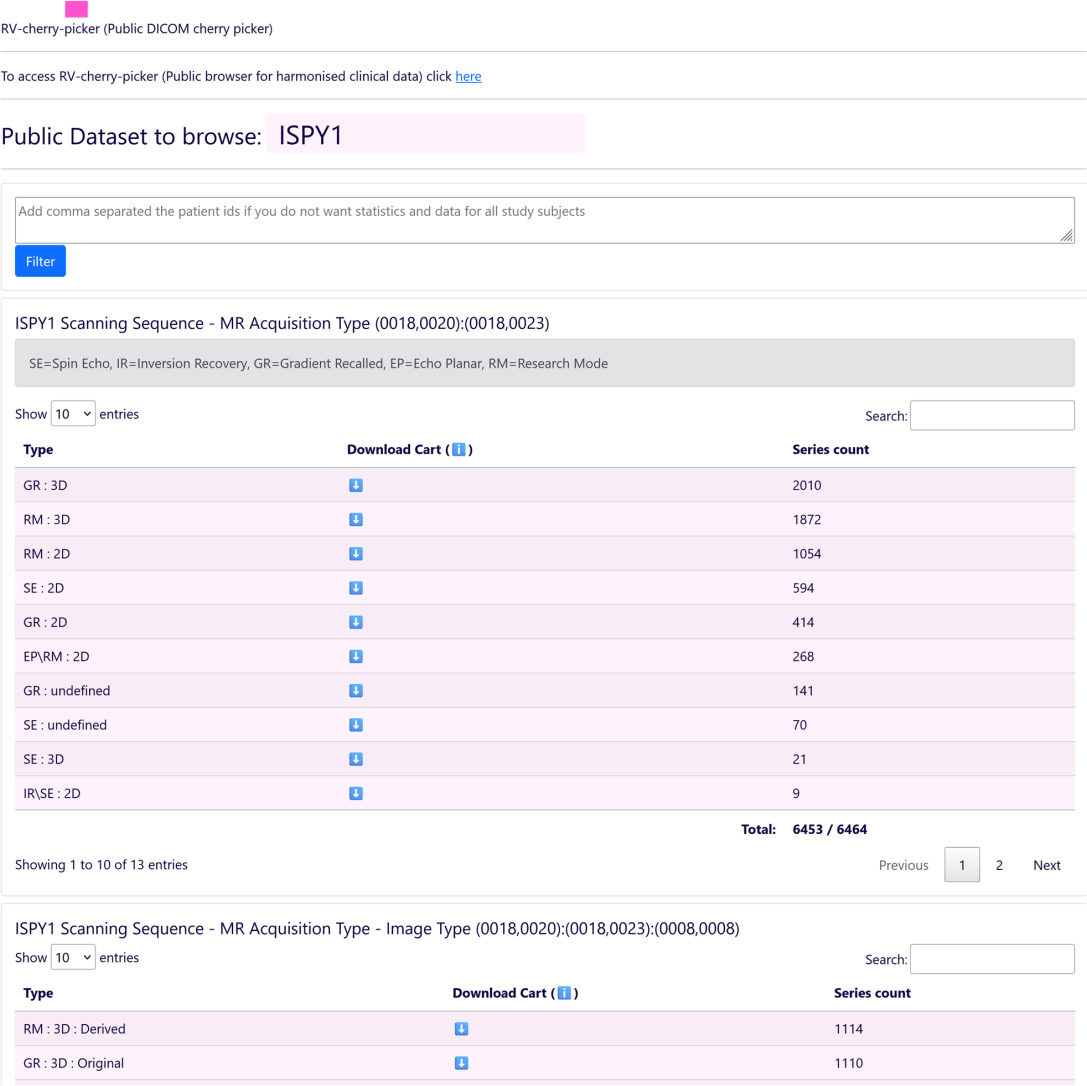
Public DICOM Cherry-Picker tool user interface excerpt

The Public DICOM Cherry Picker tool extends the existing TCIA functionality by analyzing the characteristics of each imaging dataset enabling the selection of specific subsets of the data. The selection is based on imaging technique and acquisition characteristics identified via specific DICOM tags. A part of the user interface of the tool is depicted in Fig. 4.

## Discussion

The availability of public datasets can be considered a major barrier to the production of high-quality image analysis AI systems in radiology, not only because the cost to produce these datasets is high, but also because access to existing datasets is restricted and hampered by quality problems in their curation [24]. Existing repositories, such as the TCIA and the National Cancer Institute’s Imaging Data Commons [25], offer access to both clinical and imaging data; however, most of them have significant shortcomings in terms of clinical data homogenization and deficiencies in the selection of a specific imaging subset (*e.g.*, based on specific MRI techniques).

In this paper, we focus on five datasets retrieved from the TCIA and present our efforts for creating and FAIRifying one of the most comprehensive publicly available datasets in the domain of breast cancer including both clinical and imaging data. The resulting harmonized dataset encompasses information on 2035 subjects. We detail the methodology followed, and we provide public access to the complete ETL scripts to allow both reproducibility and future dataset extension with minimal effort.

RV-Cherry-Picker allows fine-grained exploration of the dataset and enables users to search both the clinical and diagnostic imaging datasets, providing unified access and facilitating the downloading of selected series subsets, such as DWI or DCE images. This feature significantly reduces the burden of managing and identifying the appropriate sets of images for specific AI model scenarios.

Our effort enables access to the largest publicly available imaging dataset for breast cancer, already completing the first step for the development of downstream clinical AI models, *i.e.*, the step of data cleaning and harmonization, further guiding similar efforts and promoting research in the domain.

As highlighted by other authors [26], tracking and ameliorating quality problems in public datasets can have a significant impact on AI system performance. Considering this, in our future work, we plan to expand the RV-Cherry-picker platform to include compatibility checking mechanisms during the selection of imaging data originating from different device vendors or different studies, and automatically detecting curation errors and incompatibilities among the ingested datasets.

### Supplementary Information

**Additional file 1:** **Appendix.** Use case.

## Data Availability

RV-Cherry-Picker tool is publicly accessible at http://cherry.ics.forth.gr/cherry-picker/index.php. The SPSS ETL scripts are available at https://github.com/billyk18278/public-dataset-conversion-scripts. The public datasets are hosted on TCIA: • ISPY2, from https://www.cancerimagingarchive.net/collection/ispy2/. • DUKE, from https://wiki.cancerimagingarchive.net/pages/viewpage.action?pageId=70226903. • ISPY1, from https://www.cancerimagingarchive.net/collection/ispy1/. • TCGA-BRCA, from https://www.cancerimagingarchive.net/collection/tcga-brca/. • BREAST-MRI-NACT-PILOT, from https://www.cancerimagingarchive.net/collection/breast-mri-nact-pilot/.

## Abbreviations

AI: Artificial intelligence
CSV: Comma-separated values
DCE: Dynamic contrast-enhanced
DICOM: Digital Imaging and Communications in Medicine
DWI: Diffusion-weighted imaging
ETL: Extract-transform-load
FAIR: Findability, Accessibility, Interoperability, and Reuse
ID: Identifier
MRI: Magnetic resonance imaging
TCIA: The Cancer Imaging Archive

## Footnotes

1 Flip angle, the angle in degrees to which the magnetic vector is flipped from the magnetic vector of the primary field.

2 For example, in ISPY2 from the detailed definition of neoadjuvant chemotherapy medication information, both the detailed field “neoadjuvant_chemo_medication” and the binary “neoadjuvant_chemotherapy” were defined.
