# Supplementary material for: Public data homogenization for AI model development in breast cancer
Source: Eur Radiol Exp. 2024 Apr 9;8:42. doi: 10.1186/s41747-024-00442-4 (PMC11001841; doi:10.1186/s41747-024-00442-4)
Supplement: Supplementary file 1 — Additional file 1: Appendix. Use case. [file 41747_2024_442_MOESM1_ESM.docx]

## **Public data homogenization for AI model development in breast cancer**

## **ELECTRONIC SUPPLEMENTARY MATERIAL**

## Use case

As an example, use case of RV-cherry-picker, we may consider a practical scenario presenting the roadmap to achieve the goal with and without RV-cherry-picker.

**GOAL**: *Train an AI model that can identify the status of estrogen receptors (ER) of a neoplasm based on DCE MRI imaging series (using the biggest possible amount of data from TCIA)*.

To achieve the aforementioned goal, an AI developer would have to collect clinical data from various CSV/excel files provided for each dataset, identify the column referring to ER status (if this exists), and keep those subjects that have a defined value. Then recode or remap the actual value to a common value-set for all subjects/datasets. Subsequently, for each dataset, download all the corresponding imaging series and manually or programmatically open them one by one to select the series that complies with the requested imaging technique, i.e., DCE, to proceed.

With RV-cherry-picker the selection of the subject_ids and corresponding ER status is straightforward. Using Molgenis’ filters (Figure 5) all the subjects that have ER status defined are selected, easily identifying that the ISPY2 dataset does not have ER status information recorded. Then, by clicking the download button the clinical data CSV file is already coded and ready for model development.


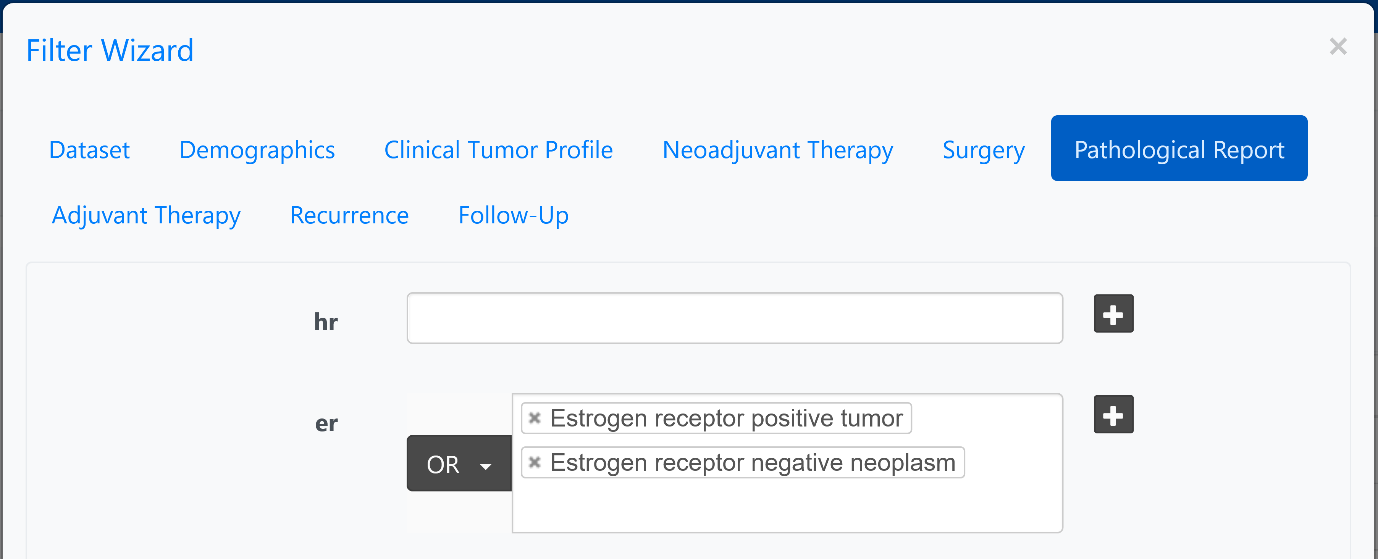


**Figure 5.** Filtering using RV-cherry-picker clinical’s filtering form for subjects with a defined value in “Status of estrogen receptors of neoplasm”.

Similarly, instead of downloading/storing 24.5K series (~1TB) of imaging data that most of which are not applicable in this scenario the developer can use the Public DICOM cherry-picker tool to skip datasets that do not have any DICOM characterized DCEs and select the images suitable for the task. This can be achieved by filtering for sequences for the specific scanning sequence and acquisition type using “Gradient Recalled” (GR) and 3D respectively, also using a filter to select original data (i.e., Imaging Type tag value starts with ORIGINAL\PRIMARY) and not to derived series that were saved after post processing. After excluding the Breast-MRI-NACT-Pilot dataset since it does not include any (GR:3D:Original) corresponding images (Figure 6.A), the developer can download, from each dataset, only the required DCE series (Figure 6.B-C-D-E).

A.
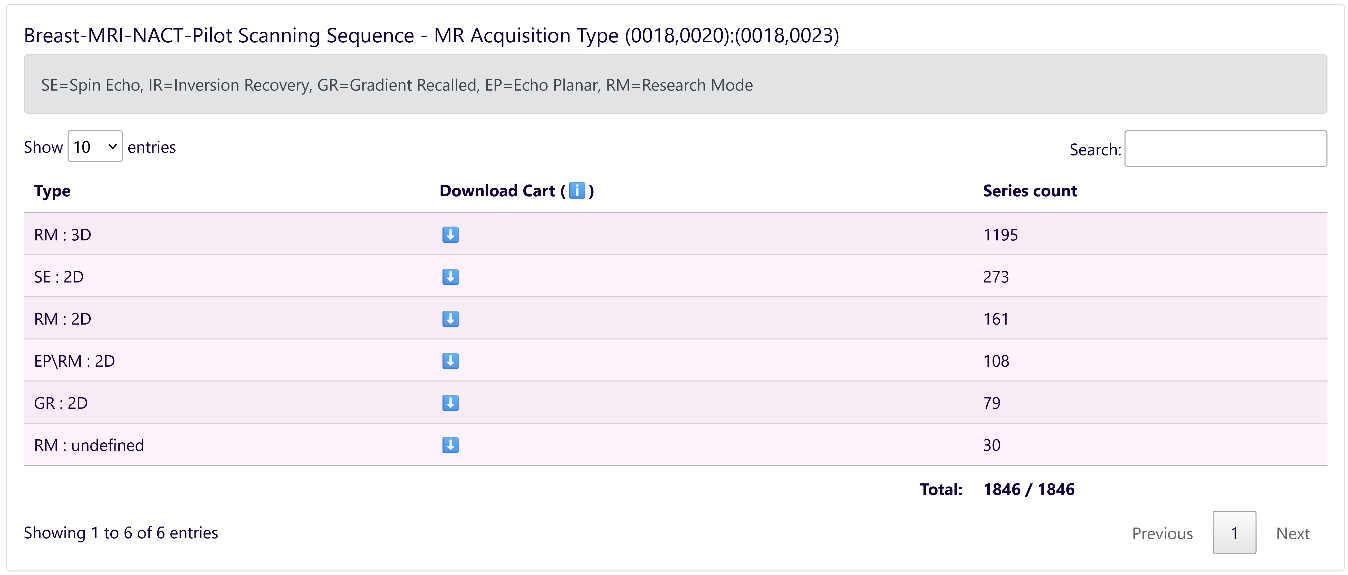


B.
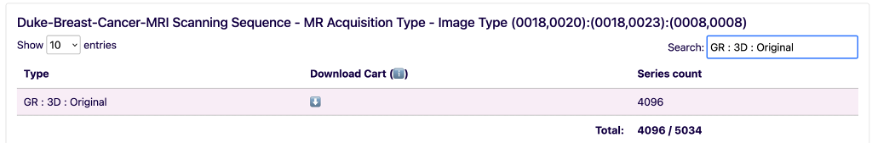


C.
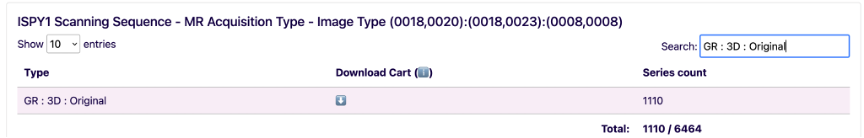


D.
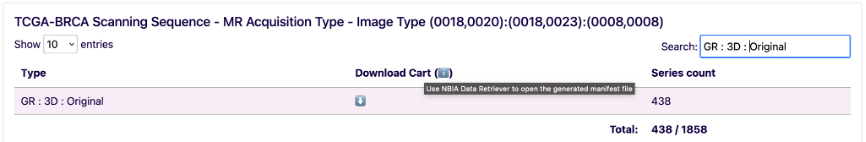


E.
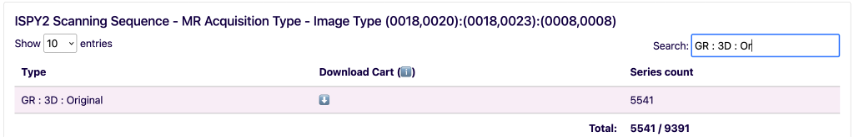


**Figure 6.** The number of MRI imaging series for the combinations of MRI Scanning Sequence and MR Acquisition Type in the five public datasets filtered to identify original DCEs (i.e. GR:3D:Original).

Apart from the significant gain of storage space by downloading only 45% (11185/24593) of the available imaging DICOM series, there is also a huge amount of effort/time saved by having the correct type of images to operate, minimizing the effort to manually inspect the selected imaging dataset. Finally, the imaging dataset may be further specialized by applying the subjects of interest as a list of IDs in the top filtering box. The list of study ids was already acquired at the first step of the process using the RV-cherry-picker clinical and it is part of the downloaded CSV file.

Although in this scenario the resulting clinical and imaging dataset acquired via RV-Cherry-picker corresponds with certainty to the requested criteria, in some datasets, additional eligible images should be present that either via anonymization or selection of different acquisition parameters might have evaded the previous selection procedure. In our example, since DCE acquisition is standard practice in the MRI protocol for breast cancer, DCEs series should be present also in Breast-MRI-NACT-Pilot. Examining the characteristics of the series from Breast-MRI-NACT-Pilot as presented in Figure 6.A, a less common practice of characterizing the scanning sequence of DCEs can be found. Specifically, instead of characterizing the sequence type as “Gradient Recalled”(GR), the characterization “Research Mode”(RM) is used. Since RM sequence type may correspond to various techniques (e.g., T2 weighted acquisitions), additional characteristics are needed to discriminate DCEs among RM series. In this case the use of filter of Flip Angle in the range of 15-30, along with 3D and Original image type can identify the DCEs and enhance the usable dataset with 195 series from Breast-MRI-NACT-Pilot (Figure 7).


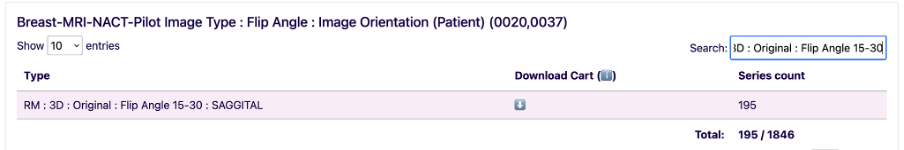


**Figure 7.** Selecting DCEs characterized as “Research Mode” (RM) by using the Flip Angle parameter.
